# Evaluating Animal-Based Foods and Plant-Based Alternatives Using Multi-Criteria and SWOT Analyses

**DOI:** 10.3390/ijerph17217969

**Published:** 2020-10-29

**Authors:** Irene Blanco-Gutiérrez, Consuelo Varela-Ortega, Rhys Manners

**Affiliations:** 1 Department of Agricultural Economics, Statistics and Business Management, ETSIAAB, Campus Ciudad Universitaria, Universidad Politécnica de Madrid (UPM), Av. Puerta de Hierro 2-4, 28040 Madrid, Spain; consuelo.varela@upm.es; 2 CEIGRAM, Universidad Politécnica de Madrid (UPM), Senda del Rey 13, 28040 Madrid, Spain; 3 International Institute of Tropical Agriculture (IITA), KG 563 Kigali, Rwanda; r.manners@cgiar.org

**Keywords:** high protein foods, meat, milk, vegetarian substitutes, innovation, diet’s sustainability, perceptions, stakeholders

## Abstract

Global diets have transitioned in recent decades with animal and processed products increasing. Promoting a reversal in these trends towards plant-based diets could reduce the environmental impacts of food systems and reduce the prevalence of non-communicable diseases and malnutrition. In Spain, a reference point for the Mediterranean diet (predominantly plant-source based), plant-based alternatives to traditional animal-based products are receiving increased attention. However, limited focus has been given to the opinions of stakeholder groups on the potential of these novel products. We evaluate the opinions of stakeholders within the Spanish agri-food sector, using multicriteria and SWOT analyses, on traditional and novel food products. Stakeholders involved in the supply chain of food products (producers, processors, and distributors) were critical of novel plant-based foods, highlighting problems with their taste, processing technology, and high prices. These results contrast with the perspectives of policymakers, researchers, environmental NGOs, and consumers who see novel products more positively - healthier, more sustainable, and highly profitable. These results illustrate the more traditional mindset seen in Spanish production systems, contrasting with the rapidly shifting tastes and demands of consumers and the potential legislative orientation of policymakers. This study calls for improved understanding and collaboration between stakeholders to better manage complex choices that affect the future of food systems during their needed transformation.

## 1. Introduction

The structure of current food systems has left more than two billion people suffering some form of malnutrition [1]. These systems are also major contributors to deforestation, soil degradation, freshwater contamination, biodiversity loss and climate change [2,3,4,5]. Increasing the sustainability of global food systems will be decisive in achieving the UN’s Sustainable Development Goals (SDGs 2, 12, 13) and stay within the boundaries of the United Nations Framework Convention on Climate Change’s (UNFCC) Paris Agreement [6]. However, sustainable improvements will have to be made in the contexts of growing global populations and rapidly evolving dietary patterns [1,6]. Food productive capacity will have to increase by at least 50% by 2050 to track increasing demand, intensifying competition for land, water, and energy [2]. These developments underscore the urgent need for policies and strategies to sustainably reorientate food systems through alternative food consumption patterns [2] and reduced environmental impacts of dietary choices [7].

Promoting healthy and sustainable food consumption presents a potential mechanism to improve the environmental condition of production systems, achieve sustainable food and nutrient security, and to enhance public health [8,9,10,11]. A number of seminal studies have outlined potential diets that, if widely adopted, could reorient food systems towards more sustainable pathways [6,12]. These studies recommend, in many cases, traditional Mediterranean or vegetarian diets as mechanisms to ensure good nutrition and reduce environmental impacts [7,9,13]. 

Despite these calls, global diets are increasingly transitioning away from basic and staple food products such as fruits and vegetables, towards higher-value (fish, meat, dairy products) and processed (high in fat, sugar, or salt) products. These trends have been implicated in the increased prevalence of non-communicable diseases and compounded the environmental health impacts of food production [1,9,14,15]. An example of where these changes have already been seen is Spain, a reference point for the Mediterranean diet, where traditional plant-based diets have become less prevalent [7,16,17]. 

Nevertheless, in several countries, including Spain, there is a small but growing number of consumers reducing, in part or completely, animal product consumption and returning to more traditional diets [17]. This segment of consumers are restricting their consumption of meat in favour of a more plant-based diet, convinced of the health, ethical, and environmental benefits of such change [18]. In response, an entire industry has grown to develop healthy and more sustainable alternatives to animal food products [19]. Manufacturers are currently focusing on new product developments, which include cultured meat and innovative plant-based foods with a high-protein content and improved texture and appearance. The European market for plant-based meat and dairy alternatives is particularly promising, with annual growth rates in of 14% and 11%, respectively [20]. The scale of growth has led to suggestions that this sector may present a step on the pathway of improving sustainable food systems [21]. 

Previous studies of novel and plant-based alternative food products to replaced animal-based products have analysed their sensory acceptance [22,23], technological feasibility [24], sustainability gains [25], nutritional and health implications [18,26], economic viability, and effects on markets and societal organisation [19,27]. However, analyses of meat alternatives as production systems remain fragmented, lacking an integrated assessment that includes their associated socio-economic, ecological, technological, and organisational implications [19]. Besides, there is a relative dearth of research regarding stakeholder responses to these products [27,28] and how they compare to the products they are designed to replace. The vast majority of scientific studies on new (plant) protein foods focus on consumers [29,30,31], neglecting the opinions of other major stakeholders directly or indirectly involved in the agri-food supply chain. The present study addresses this research gap by investigating how different stakeholders (not only consumers) evaluate plant-based meat alternative food products according to multiple value domains (economic, social, environmental, policy, sensorial, and technological). 

One method for obtaining stakeholder preferences and responses is through the use of Multicriteria Analysis (MCA). MCA provides a systematic, transparent approach that increases objectivity and can generate reproducible results by directly comparing different products, relative to each other and to specific analysis criterion [32,33]. A number of methodologies have been developed within the umbrella of MCA, including Multicriteria Mapping (MCM). MCM is used in complex and uncertain situations in which many decision factors coexist and where intuitive solutions are not easy to achieve [34,35]. MCM has been used for a wide range of different purposes, including the appraisal of options for improved food production and consumption [34,36]; health and nutrition [37,38]; energy strategy [39,40]; and environment and climate change policy consultation [41,42]. To the best of our knowledge, MCM, despite its potential, has never been used to assess stakeholders’ attitudes towards food products.

In this study, MCM will be used in combination with Strengths, Weaknesses, Opportunities, Threats (SWOT) analysis to better understand the preferences of Spanish stakeholders regarding plant-based alternatives to animal-based food products, compared to the products they are designed to replace. The analysis includes seven major groups of stakeholders (producers, processors, distributors, consumers, policy-makers, researchers, and environmental NGOs) within the Spanish agri-food sector. From this, perceived strengths and weaknesses of novel foods will be derived, as well as external opportunities and threats that may benefit or impede their further development. The study provides an integrated, structured analysis of the perspectives of key stakeholders, facilitating better-informed decisions about the future of Spanish food systems in their needed transformation.

## 2. Methodology

The present study combines a MCM and a SWOT analysis to explore how different stakeholders evaluate specific animal-based foods and plant-based alternatives. Both methods are implemented in a participatory manner, through face-to-face interviews with key stakeholders within and outside the agri-food chain, performed in 2019–2020. First, MCM is applied to examine the performance of food products according to various criteria and sub-criteria. MCM results are afterwards further applied for SWOT. A SWOT matrix is built to specifically analyse the current situation of innovative protein-rich vegetable foods and their potential to replace animal-based products. 

### 2.1. Multi-Criteria Mapping 

We use Multi-criteria Mapping (MCM) [43] to systematically clarify what food products are preferred by stakeholders and why. A key strength of MCM is the ability to capture different stakeholders’ perspectives, values, and uncertainties using structured and transparent analysis [44]. The MCM software package provides immediate visual feedback on the outcomes and choices made, enabling respondents to easily understand and modify their responses [45]. The output of MCM is both a qualitative and quantitative ‘map’ of how different options perform under distinct criteria. Although MCM can be used to explore best option, it also provides a robust and detailed understanding of how options are appraised by the respondents and how outcomes change, depending on the view that is taken and the values brought to bear in doing so [46]. 

**Figure 1:**
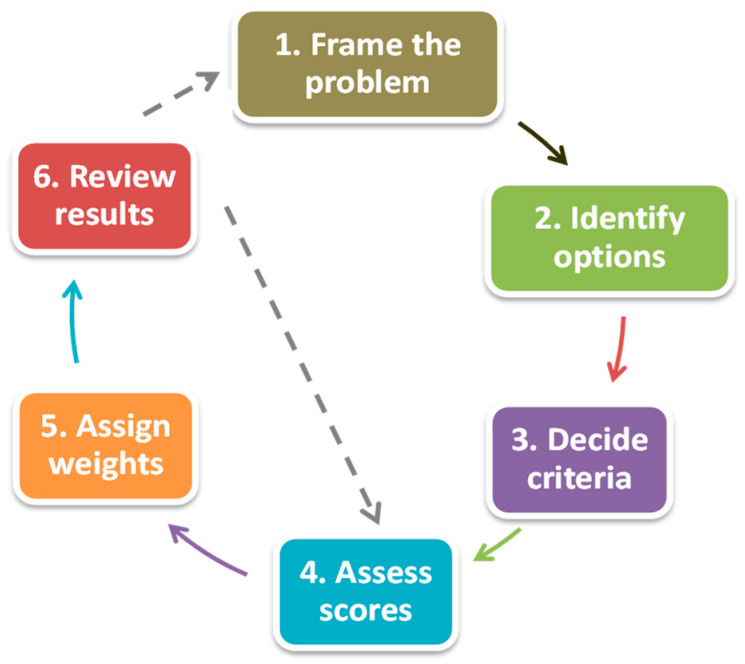
Steps of the multi-criteria mapping. Source: Adapted from Stirling [35] and Holdsworth [37].

The MCM approach usually follows six steps (Figure 1), as described by Stirling [35] and Holdsworth [37]. The following sections describe how these steps have been implemented in this study. 

#### 2.1.1. Frame the Problem

The first step in any MCM process is to frame the problem, i.e., to define the decision context and the purpose of the study. The present study has been framed inside the Protein2Food (P2F) project (Development of high-quality food protein through sustainable production and processing; Project No. 635727-2, Horizon 2020 Programme, under the Societal Challenge 2—Food Security, Sustainable Agriculture and Forestry, Marine, Maritime and Inland Water Research and the Bioeconomy, EU Commission, 2015–2020, https://www.protein2food.eu/). P2F aimed to develop innovative, healthy, and sustainable plant-based protein-rich products from multi-purpose seed crops (quinoa, amaranth and buckwheat) and grain legumes (lupin, fava bean, chickpea and lentil). Examples of such food products included plant-based milk and infant formulas, yoghurt alternatives, meat analogues, breakfast cereals, and pasta and bread products free of soy or egg proteins. With these innovative products, the project intended to increase the choice of plant-based foods and to help European consumers decrease their animal-protein intake. Inputs and feedback from key stakeholders were actively pursued and carried out through the project ‘stakeholder forum’, an advisory body to the project. The stakeholder forum met annually and sought to account for the diversity of viewpoints of EU stakeholders directly or indirectly involved in the agri-food value chain.

The present study aimed at assessing the perception of the new food products developed in P2F (and their animal counterparts) across different stakeholder groups. In total, 63 stakeholders from seven different groups (9 per group) were identified and interviewed face-to-face from 2019–2020. In our study, interviews were limited to stakeholders from the stakeholder forum who are based within Spain (geographical scope of the study) and we used snowball sampling with stakeholders to identify other individuals to interview. More information about the P2F stakeholder forum and the MCM stakeholder consultation can be found in Manners et al. [47] and Blanco et al. [48]. Stakeholder groups were defined according to the breakdown used in the P2F stakeholder forum: (1) Producers (independent farmers, livestock owners, and relevant farmers’ associations); (2) Processors (food industries, including the Small and Medium-sized Enterprises (SMEs) involved in P2F); (3) Distributors (food distributors’ associations, supermarkets, grocery stores, restaurants); (4) Consumers (flexitarians or meat-reducers, which represent the biggest growth potential for plant-based protein products [49]); (5) Policy-makers (Official Departments of Agriculture, Health and Environment at national level and the EU Commission); (6) Researchers (from P2F project and other research institutions); (7) NGOs (environmental and food security-related). 

#### 2.1.2. Identify Options (Food Products)

This step is devoted to the selection of food products to be appraised. Four food products were selected: two new plant-based products developed in P2F (lentil-based vegetable milk, and lupin and amaranth based fiber-like vegetable meat) and two animal-based products (dairy milk and chicken meat) acting as traditional counterpoints to the innovative vegetable products. Detailed information on the functional and nutritional properties of the new P2F food products can be found in [50,51].

The selection of products was based on discussions with the P2F stakeholder forum and the project team. At the time this research was carried out, vegetable milk and meat prototypes were fully developed and ready for testing. The lentil-based milk was perceived to be a good substitute for cow milk, with similar protein and fat content, and better than other typical dairy milk replacers (e.g., more environmentally friendly than soy-milk and richer in proteins than almond or rice milks) [51]. Lupin and amaranth based fiber-like meat was also considered a good alternative for chicken meat for the same reasons—its ability to provide the same amount of proteins and energy with lower environmental impact [50]. Cow milk and chicken meat were also found particularly relevant as animal-based counterpart products. Both are widespread products, commonly found in traditional European diets, that in recent years have been replaced by a growing number of plant-based substitutes. Chicken meat was chosen instead of red meat because its consumption is rapidly increasing, and its plant-based alternatives are more widely marketed and better known than beef or pork vegan products. 

#### 2.1.3. Deciding Criteria 

This step is key in any MCA. It refers to the identification of criteria, which are the measures of performance that are used to evaluate the different options (food products) previously defined. The identification process was performed in collaboration with P2F stakeholders consulted during the second stakeholder forum meeting, held in Caserta (Italy), in May 2017 [48]. The meeting was attended by 50 participants: 38 P2F project members, and 12 stakeholders from the stakeholder forum. Participants were carefully selected by the P2F coordination team, in collaboration with P2F partners and the EU Commission, to include representatives from each main group of stakeholders considered in the project and in this study (producers, processors, distributors, consumers, policy-makers, researchers, and environmental NGOs). Following Holdsworth et al. [37], the initial identification of participants was done ‘ad-hoc’, following a snowball sampling procedure. Then, high ranking individuals were selected to represent their stakeholder groups, taking into consideration their positions in terms of influence and relevance [38,52], in particular: professional knowledge and trust within their field, experience acting as representatives in relevant forums, connections to policy, capability of influencing others and capacity to engage in group discussions, level of interest and motivation in participating in the project, and capacity to disseminate the discussions and results of the project in their own field of activity. 

Participants were asked to state the most important criteria and sub-criteria that, in their view, should be considered when choosing or comparing different protein-rich food products. In total, 14 sub-criteria were chosen and grouped into 6 criteria: 1 Economic, 2 Social, 3 Environmental, 4 Policy, 5 Relevant features for the consumer, and 6 Technological. Criteria and sub-criteria are shown in Table 1.

#### 2.1.4. Assessing Scores (Scoring Food Products)

This step is devoted to the evaluation of the different food products. Stakeholders selected in Section 2.1.1, were invited to score each of the four food products (plant-based meat, chicken meat, plant-based milk, and dairy milk) on a numerical scale (0–100) under each of the 14 sub-criteria chosen (see Table 1), one-by-one, 0 representing the worst and 100 the best relative performance. These scores are not made relative to another product, but individually. A respondent is only ever considering one product at a time, so their score relative to each sub-criterion is independent of all other products. To allow uncertainty in the estimation, the MCM software allows respondents to give a range (‘pessimistic’ or minimum score, and ‘optimistic’ or maximum score) rather than a single number [45]. During the scoring process, stakeholders were asked to explain the range assigned to the different food products and the assumption made when deciding value ranges. Questions used to score each food products under different sub-criteria are shown in Table 1. 

#### 2.1.5. Assigning Weights (Weighting of Criteria and Sub-Criteria)

In this step, respondents assign weights to each criterion and sub-criterion. We utilised a methodology similar to Stirling and Mayer [34], Hansen [53], McDowall and Eames [39], and Raven et al. [42] to assign weights. Firstly, stakeholders were asked to assess the importance of the different criteria when choosing food products, with a single score (not a range) from 0 (representing the worst) to 100 (the best), so the more points they gave to a criterion the more important it was considered. These scores are not made relative to another criterion, but individually (each criterion can be given 100). Secondly, stakeholders were invited to evaluate the importance of each sub-criterion with respect to the criterion it belongs to. Stakeholders were asked to distribute the score given to a criterion among all the sub-criteria that were part of this criterion. 

#### 2.1.6. Reviewing (Examining) the Results

The MCM software provides a series of parameters to facilitate the interpretation and review of results. In the present study, the following parameters were used:

“Ranks”. This is used to calculate the overall performance rank for each food product, under all the criteria and sub-criteria taken together for a specific stakeholder’s perspective. The MCM software uses the simple ‘linear additive weighting’ method, in which ranks are derived by adding weighted scores. The minimum (‘pessimistic’) and maximum (‘optimistic’) scores given by a stakeholder to a product under a specific criterion are multiplied by the weight percentages assigned to that criteria. Ranks are calculated by repeating this process for each criterion and summing all values obtained. The final ranks of products were discussed in-situ with stakeholders to check whether they accurately represented their perspective. 

“Uncertainty”. This parameter reflects the degree of uncertainty displayed in the scores and resulting ranks of interviewees. In other words, it reveals how stakeholders express how unsure they are in their scoring intervals. We use ‘ratio uncertainty’, which is expressed as a ratio to the median score. A high score means a high degree of uncertainty, and on the contrary a low score means a low degree of uncertainty. The degree of uncertainty assigned to a specific stakeholder group is represented as a mean value for all respondents (stakeholders) belonging to the group. 

“Weights”. This parameter displays the relative magnitudes of weightings assigned to different criteria under different stakeholder’s perspectives. The MCM software only shows the range of weights attached to criteria; it does not provide information about the weights of different sub-criteria. For that reason, the weight that each sub-criterion has concerning the criteria group it belongs to has been calculated ex-post, not with the MCM software.

For further information about the arithmetic behind the calculation of ranks, uncertainties, and weights, see Annex A of the MCM manual [45]. Furthermore, we calculated two additional parameters (sub-criteria weighted score and criteria weighted score) to analyse in more detail the performance of food products under different criteria and sub-criteria. 

“Sub-Criteria weighted score” (SubWS) of a food product *p*, for a stakeholder *i*, is represented by the following equation:

(1) $$Sub\;WS_{p,i}=\bar{scr_{p,i}}\cdot \frac{Wsub}{\sum_{r}Wcrt_{r}}$$

where ($\bar{scr_{p,i}}$) is the average score value of a product *p* for a stakeholder *i* (the average of minimum and maximum scores given by a stakeholder to a product under a specific criterion), *Wsub* is the weight of the sub-criteria, and *Wcrt_r_* is the sum of weights assigned to each of the criteria groups *r*. The sub-criteria weighted score shows the performance of food products under specific sub-criteria and individual stakeholder perspectives. The value for a group of stakeholders is calculated by averaging scores across stakeholders belonging to the group. 

“Criteria weighted score” (CritWS), of a food product *p*, for a stakeholder *i*, is obtained as shown in the following equation:

(2) $$Crit\;WS_{p,\;i}=\underset{s}{\sum }Sub\;WS_{p,i,s}$$

where *Sub WS_p,i,s_* is the sub-criteria weighted score of a food product *p*, for a stakeholder *i*, and a sub-criteria *s*. Therefore, the criteria weighted score is simply the sum of all the sub-criteria weighted scores that are included in a group of criteria. This shows the performance of food products under specific criteria and individual stakeholder perspectives. Again, the value for a group of stakeholders is calculated by averaging scores across stakeholders belonging to the group. 

### 2.2. SWOT Analysis

SWOT is a well-known method used by companies for strategy formulation and development [54]. It stands for ‘strengths, weaknesses, opportunities, and threats’. Strengths and weaknesses are internal factors that offer the products a competitive advantage or disadvantage. On the contrary, opportunities and threats are external factors capable of facilitating or hindering the development of a product. Recently, several studies have attempted to use SWOT for the entire supply chain in agricultural product strategy formulation, such as Suwanmaneepong et al. [55] who used SWOT to develop marketing strategies for agricultural products. Baudino et al. [56] also applied SWOT to analyse the environmental impact of different food production supply chains.

In the present study, we performed the SWOT analysis as a complement to the MCM analysis. At the end of each of the interviews, each stakeholder was asked to describe the strengths, weaknesses, opportunities, and threats for each food product. Following Prišenk and Borec (2012) [57], MCM results were used as a base for SWOT. Those MCM criteria and sub-criteria that rated the highest were considered potential strengths of the products. On the contrary, those criteria and sub-criteria that received the worst rating were deemed as potential weaknesses. Considering the objective of the P2F project (developing innovative protein-rich vegetable products for replacing animal-based products), we focused on plant-based products to try to investigate the trends and patterns that may have either positive or negative impacts on the development of modern vegetable meat and milk alternatives. Results from interviews were used to build a SWOT matrix, in which strengths, weaknesses, opportunities, and threats of plant-based products were examined and differentiated by stakeholder groups. 

## 3. Results

### 3.1. Ranking of Food Products

**Figure 2:**
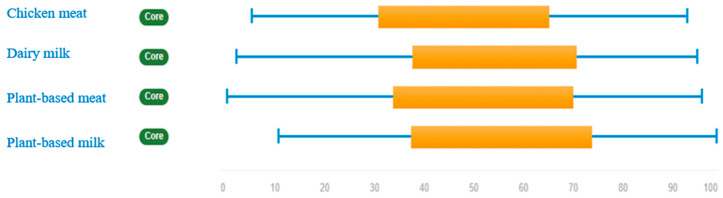
Overall ranking of food products (all stakeholder’ views). Solid orange bars show rank means (the left and right ends indicate the means of the low and high ranks assigned by each stakeholder). Thin blue lines refer to rank extrema (the left and right ends indicate the lowest and highest ranks assigned by any stakeholder).

Figure 2 shows the rank values of the four food products examined under all stakeholder’ views. Results indicate that the most preferred product is plant-based milk (which has the highest mean and extrema scores), followed by dairy milk, and plant-based meat. The least preferred food product is chicken meat. Nevertheless, if we compare vegetable-based products against animal-based products, it can be observed that the former are preferred over the latter (i.e., plant-based meat is preferred to chicken meat, and plant-based milk to dairy milk). Both pessimistic (low) and optimistic (high) mean scores (left and right sides of the orange bar, respectively) are greater for vegetable-based products than in animal-based products. Only in the case of milk is the pessimistic mean score similar for dairy milk and plant-based milk. 

**Figure A1:**
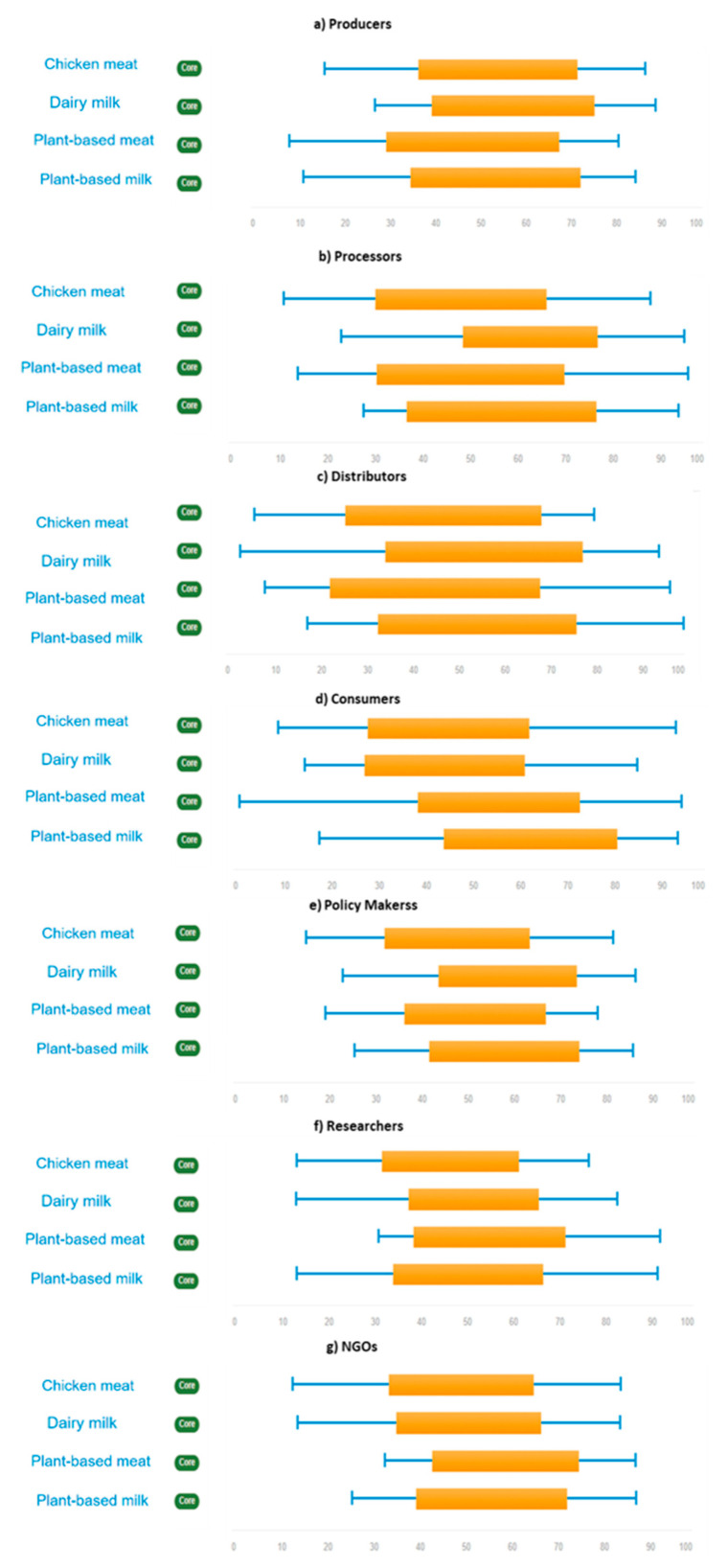
Overall ranking of food products by stakeholder group: (**a**) Producers; (**b**) Processors; (**c**) Distributors; (**d**) Consumers; (**e**) Policy-makers; (**f**) Researchers; and, (**g**) NGOs e. Solid orange bars show rank means (the left and right ends indicate the means of the low and high ranks assigned by each stakeholder). Thin blue lines refer to rank extremes (the left and right ends indicate the lowest and highest ranks assigned by any stakeholder).

Looking at each stakeholder group individually (Figure A1), the analysis shows that consumers, researchers, and members of NGOs have the highest opinion (i.e., rank values closer to 100) of vegetable products. Only researchers assigned higher pessimistic scores to dairy milk than plant-based milk. In contrast, interviewees within the agri-food supply chain (processors, distributors, and notably producers) prefer animal-based products than vegetable-based products. In general, they assign higher mean scores to products of animal origin, except in the case of meat for processors, where chicken meat has a lower optimistic score than vegan meat. Finally, policymakers do not show a clear preference for vegetable products or animal-based products. They rank dairy milk higher than vegan milk, but rank chicken meat lower than vegan meat. 

### 3.2. Uncertainty

**Figure 3:**
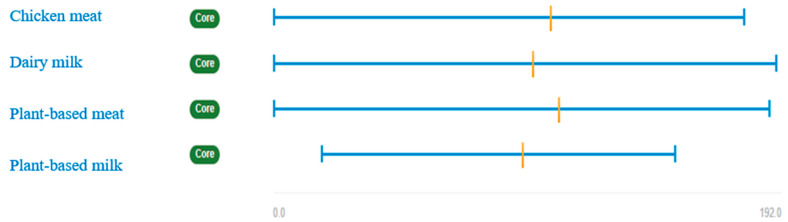
Uncertainty (all stakeholder’ views). The orange cross-line represents the average degree of uncertainty. The blue line refers to extreme values (the left and right ends show the largest and lowest degrees of uncertainty).

Figure 3 displays the uncertainty attached to each product according to all stakeholder’s views. Results show that plant-based meat has the highest average degree of uncertainty (the orange cross-line is located more to the right), followed by chicken meat, and dairy milk. Plant-based milk shows the lowest average degree of uncertainty. These findings indicate that meat products have more uncertainty (stakeholders are less sure about their scores) than milk products. Furthermore, no conclusive results are obtained when animal-based products and vegetable products are compared. Plant-based meat has a slightly higher degree of uncertainty than chicken meat, but dairy milk shows more uncertainty than plant-based milk. 

**Figure A2:**
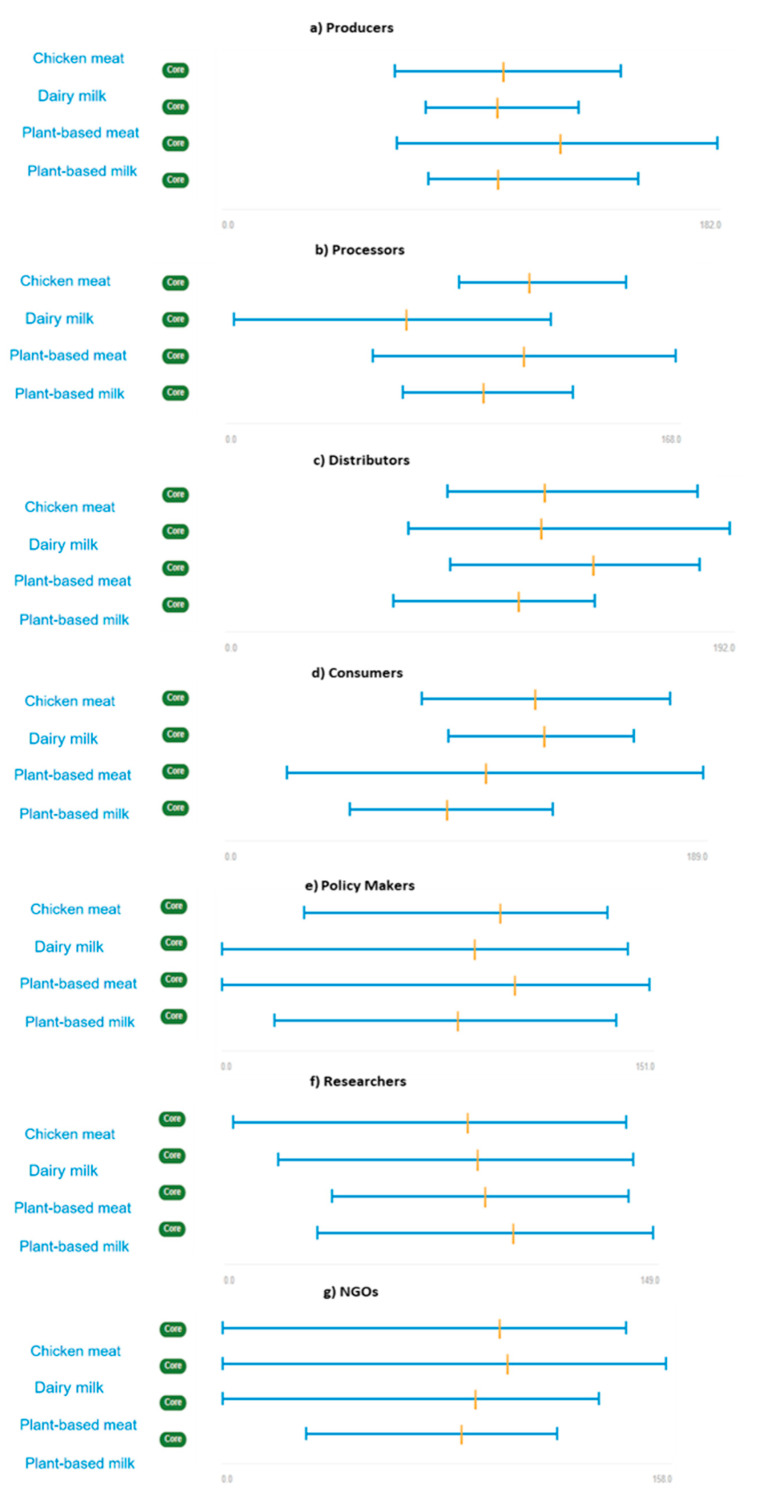
Uncertainty by stakeholder group: (**a**) Producers; (**b**) Processors; (**c**) Distributors; (**d**) Consumers; (**e**) Policymakers; (**f**) Researchers; (**g**) NGOs. The orange cross-line represents the average degree of uncertainty. The blue line refers to extreme values (the left and right ends show the largest and lowest degrees of uncertainty).

Looking at the breakdown by each stakeholder group (Figure A2), results show that plant-based meat is the product with the highest average degree of uncertainty for producers, processors (together with chicken meat), distributors, and policy-makers. Dairy milk is the most uncertain product for consumers and members of NGOs, and plant-based milk for researchers. 

Results also demonstrate that for both plant-based and animal-based products, members of NGOs, policy-makers, researchers, and consumers were more decisive in their answers than producers, processors, and distributors. The orange bars (Figure A1) are shorter (differences between pessimistic and optimistic scores are smaller) in these groups. Furthermore, the left side of the blue line (lowest uncertainty) in Figure A2 is 0 for some products in the case of policy-makers, researchers and environmental NGOs, which means that some stakeholders belonging to these groups are extremely sure about their answers. This does not happen among producers, processors, and distributors, who responded with an ample range of uncertainty. 

### 3.3. Criteria and Sub-Criteria Weights

**Figure 4:**
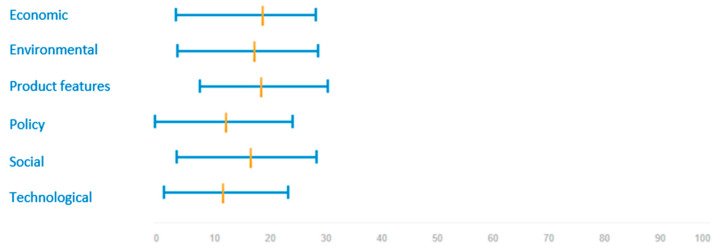
Weights assigned to each criterion (economic, environmental, features for consumers, policy, social, technological) (%) (all stakeholders’ views). The orange cross-line shows the mean value. The blue horizontal line shows the range between lowest and highest weights attached to each criterion.

Figure 4 shows the weights assigned by all stakeholders to each of the six criteria examined: economic, environmental, product features relevant for consumers, policy, social, and technological. 

Results show that the most important criterion when choosing a food product according to all stakeholder’s views is economic (19.6% mean weight), followed closely by product features (flavour and cooking properties, 19% mean weight), environmental (18.2%) and social (17.4%). The lowest weighted criteria are policy (13.1%) and technological (12.7%). 

**Figure A3:**
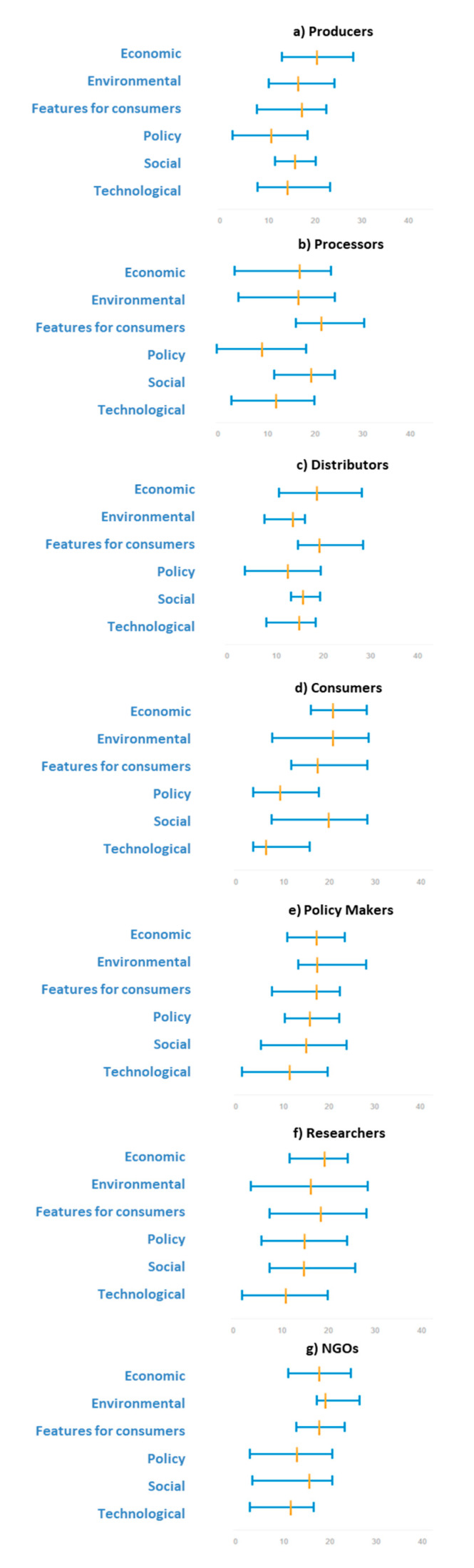
Weights (%) assigned to each criterion by stakeholder group: (**a**) Producers; (**b**) Processors; (**c**) Distributors; (**d**) Consumers; (**e**) Policymakers; (**f**) Researchers; and (**g**) NGOs.

Differences among criteria are more evident when looking at the weights assigned by each stakeholder group (see Figure A3 and Table A1). Results indicate that for the stakeholders within the agri-food supply chain (producers, processors, distributors, and consumers), economic and product features are the most important criteria. While producers, consumers and researchers give more importance to economic criteria, processors and distributors value product features more (e.g., flavour and cooking properties). Stakeholders with a high degree of environmental awareness, such as members of NGOs and policy-makers, give more weight to environmental issues. The social criterion is middle ranked in all stakeholder groups. Finally, policy is the least weighted criterion for producers, processors, and distributors, while technological is least weighted for consumers, policymakers, researchers, and environmental NGOs. 

**Figure 5:**
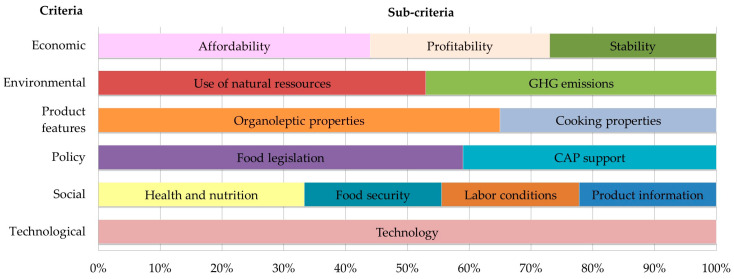
Weights assigned to each sub-criterion (% of the total weight of the criteria) (all stakeholder’ views).

Weights of the different sub-criteria are shown in Figure 5 (all stakeholder’ views together) and Table A1 (by type of stakeholder group). Results indicate that, for all stakeholders, affordability is the most important economic sub-criterion (representing 44% of the total mean economic weight), followed by stability and profitability (both approximately 27%–29%). Regarding the environmental domain, the most important sub-criterion is use of natural resources (53% of the total mean environmental weight), especially for producers (56%), distributors (59%), and policymakers (56%). Only researchers score emissions related to climate change slightly higher emissions than use of natural resources 51% versus 49%). In features relevant for consumers, all groups of stakeholders (notably distributors) give much more weight to organoleptic properties (e.g., flavour) than they do to cooking properties. On average, organoleptic properties represent 65% of total features’ mean weight, while cooking properties only represent 35%. Concerning policy issues, food legislation is the sub-criterion with most weight, counting for 59% of the total mean policy weight. Processors (63%), distributors (72%) and policymakers (67%) give food legislation the highest weight (63%, 72%, and 67%, respectively). Only members of NGOs value Common Agricultural Policy (CAP) support more than food legislation (53% versus 47%). Regarding social criteria, health and nutrition is the most important sub-criterion (34% of the total mean social weight), notably for distributors (36%), policymakers (42%), and researchers (37%). The other three social sub-criteria (food security, labour conditions, and product information) are weighted similarly (22% each). Finally, concerning technology, no sub-criteria were defined. Distributors are the group that value it the most and consumers the least (see Table A1). 

### 3.4. Performance of Food Products

**Figure 6:**
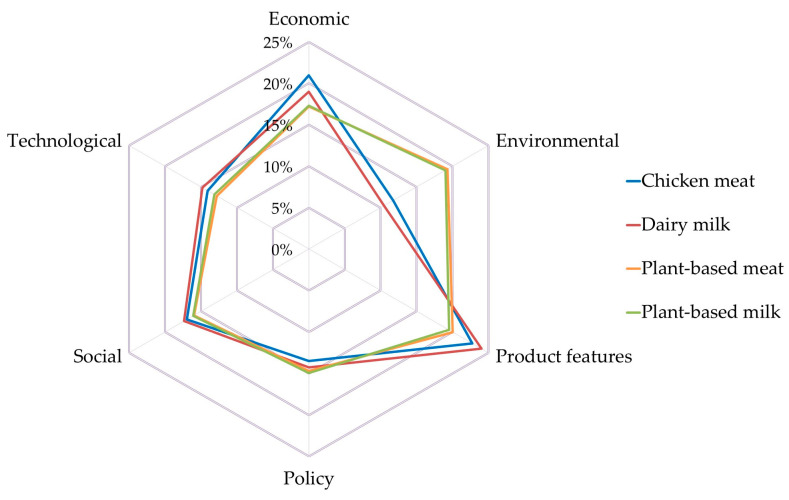
Overall performance of food products under each criterion (all stakeholder’ views). Average weight of each criterion (%).

**Figure 7:**
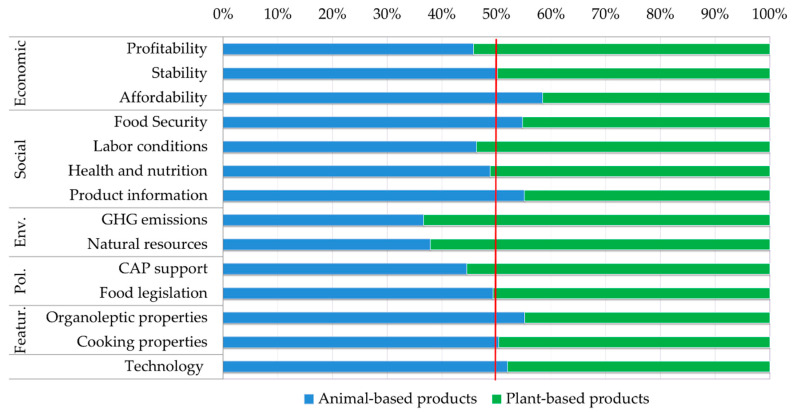
Overall performance of food products grouped into animal-based and plant-based products, under each sub-criterion (all stakeholder’ views). Average weight of each sub-criterion (%).

The performance of food products has been analysed examining the results of the ‘Criteria weighted score’ (CritWS) (Figure 6 and Table A2) and ‘Sub-Criteria weighted score’ (SubWS) (Figure 7 and Table A3). Figure 6 shows the overall performance of food products under each criterion (i.e., how all stakeholders value different products under each criterion). Disaggregated results by group of stakeholders are displayed in Table A2. 

Results indicate that the weight of all criteria is almost the same in the two plant-based food products analysed (meat and milk) (see Figure 6). In both products, the environmental and product features criteria are weighted the highest (19%–20% on average), followed by economic (17%), social (16%), and policy (15%) criteria, and finally the technological criterion (13%). Table A2 shows that consumers, policy makers and members of NGOs, particularly value the environmental performance of these products (they assign the highest weights to the environmental criterion, about 20%–24%), while processors, distributors, and researchers give more importance to product features (20%–25%). Here again, the differences between plant-based meat and plant-based milk are small. In the case of producers, both criteria (environmental and product features) are weighted similarly (19%–20%), closely followed by the economic criterion (17%–19%). All stakeholders weight least the technological criterion (only 6%–7% in the case of consumers), except producers and processors, who assign the lowest score to the policy criterion (11%–13%). 

Furthermore, the results obtained, shown in Figure 6, indicate that criteria in the two analysed products of animal origin are also weighed similarly (although more differences can be found between chicken meat and dairy milk than between plant-based meat and plant-based milk). Product features weigh the highest (23%–24% of the total), closely followed by economic (19%–21%) and social criteria (17%). Technological and policy criteria have almost the same weight (14%–15% and 13%–14%, respectively). The environmental criterion is weighted least (around 10% for dairy milk and 12% for chicken meat). Processors, distributors, policy makers, researchers, and members of NGOs give the highest weight to the product features criterion (20%–26%), notably when it comes to dairy milk (see Table A2). Producers, and notably consumers, value more highly the economic criterion (18%–25%), particularly in the case of chicken meat. For all stakeholder groups, the social criterion is also important. It is weighted highly (15%–21%), slightly below the economic criterion. As in the case of plant-based products, the least weighted criterion for producers and processors is policy (12%–13%), while for consumers it is technological (9%–10%). For the rest of the stakeholder groups (distributors, policy makers, researchers, NGOs) it is the environmental criterion (9–12%). 

The largest differences in criteria weights are seen between animal-based and plant-based products (Figure 6). On average, products of animal origin show a better performance than products of plant origin in the following criteria: product features (weighted 3%–5% higher), economic (+2%–4%), technological (+1%–2%), and social (+1%–2%). On the contrary, plant-based products have a better performance than animal-based products under environmental (+7%–9%) and policy (1%–2%) criteria.

Looking at sub-criteria level (Figure 7), we can see that only 5 (out of 14) sub-criteria have higher weights in products of animal origin than in products of plant origin: affordability (weighted 58% in animal products vs. 42% in vegetable products), food security, product information and organoleptic properties (55% vs. 45%), and technology availability (52% vs. 48%). Animal-based products and plant-based products have similar weights concerning stability and cooking properties (50% vs. 50%). Finally, plant-based products are perceived as having better performance than animal-based products in 7 (out of 14) sub-criteria: profitability (54% vs. 46%), labour conditions (54% vs. 46%), CAP support (55% vs. 45%), health and nutrition and food legislation (51% vs. 49%), and notably GHG emissions (63% vs. 37%), and natural resources use (61% vs. 38%).

As can be seen from Table A3, all stakeholders agree that plant-based products are more profitable and less affordable (final prices are higher) than animal-based products. Consumers and NGOs also perceive that plant-based products face lower risks (i.e., less variability of prices and yields) than animal-based products, and therefore believe that they are more stable. Only distributors think the opposite. All other stakeholders consider that both products are equally stable. 

Regarding social issues, most stakeholders assign higher weights to food security for animal-based products than in plant-based products. Consumers are the only group that believe that more and better proteins can be obtained from novel plant-based products. Furthermore, all stakeholders (except producers and processors) perceive products of plant origin to be healthier and more nutritious than those of animal origin. However, they think that product information is more adequate in the case of products of animal origin. 

Reactions also diverged regarding policy aspects. While producers and distributors think that policy support (CAP subsidies) is more convenient in the case of animal-based products, other stakeholders think the opposite. 

The strongest consensus is found around environmental issues. In all stakeholder groups, the weight assigned to (low) GHG emissions and use of natural resources is higher in plant-based products than animal-based products. 

Concerning features relevant for consumers, all stakeholders agree that animal-based products are tastier and more pleasant than plant-based products. Consumers and processors also think that animal-based products are easier to cook. Finally, there is also a consensus that the production and processing technology of innovative plant-based foods has still room for improvement. Most stakeholders assign higher weights to technology in animal products than in vegetable products.

### 3.5. SWOT Analysis

**Figure 8:**
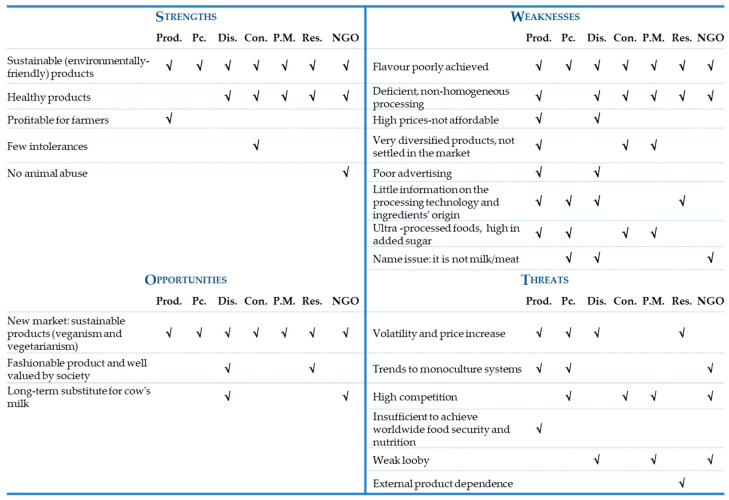
Strengths, Weaknesses, Opportunities, Threats (SWOT) analysis of innovative protein-rich plant-based products. Stakeholder groups: Prod. (producers), Pc. (processors), Dis. (distributors), Con. (consumers), P.M. (policy-makers), Res. (researchers), NGO (non-governmental organization).

Figure 8 shows the responses of all stakeholder groups regarding the strengths, weaknesses, opportunities, and threats of innovative protein-rich vegetable products (plant-based milk and plant-based meat). 

SWOT results show that stakeholders tend to see more weaknesses than strengths in relation to plant-based food products. Most of them agree that flavour is poorly achieved and that the processing of food is not homogenous, even deficient. Information on processing technology and the origin of ingredients is also considered poorly reported. Some stakeholders indicate that these products are too diversified and not well settled in the market. For producers and distributors, advertising is poor and sometimes confusing (traditional labelling like ‘meat’ is not allowed for plant-based foods). In some cases, they can convey the image of being ultra-processed products, with a high added sugar content. For all stakeholder groups, the greatest strength of these products is that they are sustainable (environmentally friendly products). Many stakeholders also think that they are healthy products. 

Concerning external factors, stakeholders also tend to see more threats than opportunities. However, there seems to be little consensus about threats among stakeholders because they provided much more dispersed answers. The threats most mentioned are the impact of high and volatile prices of agricultural food commodities, the strong competence of other food products in the market, the growth of monoculture crops (e.g., wheat) which can make legumes less attractive and, lastly, the relatively weak lobby for plant-based foods (notably less strong than, for example, the dairy lobby in the EU). Concerning opportunities, all stakeholders agree that the main opportunity for these two innovative products (plant-based milk and plant-based meat) is the big new market that is emerging, that is to say, a new market space for flexitarian or vegetarian consumers who also buy with environmental awareness.

## 4. Discussion

We implemented a mixed methods approach (MCM and SWOT) to better understand the perspectives of different stakeholder groups on novel (plant) and traditional (animal) protein food products in Spain. This methodology has been found valuable but has some limitations, particularly in relation to the application of the MCM software. MCM emphasises interaction with stakeholders, obtaining as much qualitative and in-depth information as possible, but it is less detailed than other MCA methods in the way weights and group preferences are evaluated. Possible extensions of the MCM method could consider more sophisticated methods of determining weights (see e.g., Azadfallah [58] or Stanujkic et al. [59]), and different approaches to aggregate preferences, when the objective is to reach consensus and decision makers have different experiential, cultural and educational backgrounds (see e.g., Chen [60]). 

Results show divergent perspectives on these food products across the stakeholder groups. These opinions could be differentiated based upon whether stakeholders were involved (or not) in the production process. We found that stakeholders not involved in the production side (consumers, policy-makers, researchers, and members of NGOs) consistently ranked novel products higher, contrasting with producers, processors, and distributors, whose responses were more uncertain. 

We found that novel plant-based products are perceived as being more profitable and capable of providing improved labour conditions than animal-based products. However, this is seen as an opportunity created by the rapidly growing market fuelled by flexitarian and vegetarian consumers more than as a strength, evidencing that the economic benefits of novel food products are still not very well understood. 

Falcone et al. [61] evidence the lack of socio-economic assessment of plant-based alternatives, which contrasts with the high importance placed upon economic considerations by stakeholders. Our study reveals that economic considerations are the priority when choosing food products, closely followed by product features, environmental and social (health) issues. This would suggest the need for greater emphasis to be placed on the economics of novel plant-based food products in their roll out to producers and consumers. 

Our findings on price (affordability), organoleptic properties, and use of natural resources, as the three key considerations of consumers, contrasts with those of previous studies where health benefits were found to be the main motivator for food product selection (and particularly for meat reduction) [29,31,62].

The fact that environmental issues are considered more strongly than health and nutrition-based considerations is interesting. As reported in previous studies [7,63], this could be an indication that awareness of the environmental impacts of food consumption is increasing within Spanish society. In our study, this is considered a great opportunity to introduce more environmentally friendly foods, like protein-rich plant-based products, in Spanish diets. Results indicate that interviewed consumers, researchers, policy-makers, and NGO groups highly valued plant-based food products because of their positive environmental impacts and health and nutritional benefits and highlight these two aspects as the main strengths of novel (plant) protein foods. This suggests that these stakeholder groups may be more conscious of the potential benefits of these replacement products and also of the importance of a shift towards more sustainable consumption patterns in Spain. This assertion would support the general idea that a growing number of consumers are aware of the health, ethics, and environmental impacts of current diets [18,26,27], and contrasts with the ideas of Hartmann and Siegrist [64] and Happer and Wellesley [65] who found that consumers are in general unaware of the environmental impacts of meat consumption and that the willingness to reduce or substitute meat is still very low. 

The positive scores given to novel plant-based products, particularly by consumers, appear to support the nascent shift in consumption patterns away from animal based products previously observed in Spain and other countries [17,30,66]. However, our study suggests that this shift could be implemented in Spain at a slower pace than expected, given the high number of weaknesses and threats still perceived in relation to novel food products. In line with these findings, Zarbá et al. [67] reported that the adaptation of novel food in EU member states bordering the Mediterranean Sea will probably occur later than in other member states with less deeply rooted cultural traditions and a more open attitude to innovation and experimentation in the food sector.

Our results indicate that producers, processors, and distributors, those involved in the production side, identified more weaknesses and threats than strengths and opportunities for alternative plant-based products. These findings illustrate the more traditional mindset seen in Spanish production systems, and contrast with the rapidly shifting tastes and demands of consumers and the potential legislative orientation of policymakers. 

The major weaknesses of plant-based food products identified in our study are high consumer prices, lack of similarity in taste and texture to traditional meat and dairy products, drawbacks in the development and application of new processing technologies, and limited product information in terms of labelling and marketing. These findings reinforce the idea that the widespread consumption of plant-based foods may be minimal if these products continue to be perceived as excessively expensive in comparison to traditional animal-based products [47]. In this sense, numerous studies have reported that high price is one of the main barriers to buying sustainable products [30,68], and also to substituting meat with novel plant-based alternatives [28,69]. Manners et al. [47] state that increasing transparency in pricing and other market information in the supply chain of plant-based products would allow different actors to make more informed choices and contribute to strengthening the position of producers and primary processors in the food supply chain. The stakeholders interviewed in the present study indicated that improved information about the origin and processing of novel products in labelling and marketing could help increase consumer confidence and convey their proven environmental and health benefits to the public. Additionally, Apostolidis [30] reported that policies and strategies need to move beyond providing information, facilitating consumers’ empowerment through effective price-based strategies and consumer education. 

Furthermore, our results support previous findings that new plant-based products continue to create doubts about taste and processing [70]. Mancini and Antonioli [71] found that consumers are more positive towards the extrinsic (environmental performance) than intrinsic (taste and processing) attributes of meat alternatives. Also, Weinrich [72] mentioned that improving the appearance and taste of meat substitutes would be crucial to promote their consumption on a regular basis. 

As with taste and processing issues, the highly-processed nature of many plant-based food alternatives remains a concern. Broad [21] indicates that this concern generally focuses on questions of health and food safety. In addition, van der Weele et al. [19] and Smetana et al. [25] state that high levels of transformation and processing may limit the environmental sustainability gains of plant-based meat alternatives and therefore their uptake by the most environmentally conscious consumers. Additionally, the stakeholders interviewed in the present study raised doubts about the adequacy of current production and processing technologies. This behaviour is in line with the research made by Aiking [73] and Aydar et al. [24], who reported an insufficient technological know-how about novel (plant) protein foods and called for disruptive technological innovations to make these novel alternatives viable. 

However, some studies argue that these technological improvements should be accompanied by organisational and institutional changes in order to be effective [19]. In addition, we suggest that better understanding and collaboration among stakeholders could contribute to accelerate these changes. As previously reported by Manners et al. [47], our study highlights that greater policy and financial support along the entire value chain would be essential to foster the plant-based food business. The recently launched Farm to Fork EU strategy and the new EU Common Agricultural Policy (2021–2027) offer great opportunities to promote the plant-based protein sector in EU countries, including Spain, by outlining a new integrated food policy devoted to making agricultural and food practices more sustainable.

## 5. Conclusions

A mixed method approach (MCM and SWOT) was implemented to evaluate the perception of different stakeholders regarding traditional animal-based and novel plant-based foods in Spain. The combination of MCM and SWOT analyses (both based on qualitative assessments) has clear synergies and has resulted in an efficient practical planning tool for encouraging the expansion of plant-based proteins. The SWOT analysis was supported by the MCM results (most important criteria and sub-criteria) that were then classified in a structured way. This has permitted us to represent the shortcomings and potential developments of novel plant-based foods more clearly and transparently. The joint results of MCM and SWOT analysis provide a good information base for policy-makers as to which aspects need to be improved for the successful expansion of novel plant-based foods in Spain. 

This study has demonstrated the dichotomous perception of novel meat alternative products across the agri-food system in Spain. Stakeholders involved directly in production or processing perceive these products poorly, compared to consumers, researchers, members of NGOs, and legislators. Producers, processors, and distributors see greater weakness of and threats to novel plant-based alternatives, compared to animal-based products. The more favourable perspective of consumers and other groups may be a clear indicator of a shift in Spanish consumption patterns back towards healthier and more sustainable diets, and that plant-based food alternatives are increasing in popularity, mainly because of health, ethics, and environmental concerns. Our findings contrast with previous studies and stress that economic and taste still outweigh environmental or health considerations for consumers when it comes to product selection, suggesting the need for price parity before widespread consumption becomes the norm. However, with parity, the importance of environmental concerns for consumers may suggest that one of the main drivers of nascent potential dietary shift is rising environmental awareness. To ensure greater consumption and public acceptance our results also suggest that plant-based products should improve their processing and origin information, have more affordable prices, and improve their flavour.

Our study concludes that the growth in the plant-based products sector in Spain could be lower than expected unless production and information improve significantly in years to come. These improvements would require significant policy support along the entire value chain, currently perceived as necessary, but not yet sufficient to foster plant-based business. New policies and reforms are needed to support the labelling and production of vegetable products and convince producers of their value. However, the success of these policies and strategies lies largely in the actions of and acceptance by stakeholders. 

This study provides useful information on the perceptions of different Spanish stakeholders regarding the development of plant-based food products. Such information will help potential plant-based food developers (food industry and scientists), and in particular decision-makers, to take better informed decisions on the promotion and adoption of plant-based food patterns in Spain, and to facilitate the uptake of these decisions. 

Our study analyses the opinions of the different stakeholders individually. Further studies should consider multi-stakeholder processes to facilitate debate and discussion among the most concerned actors and promote collaborative stakeholder efforts. New multi-stakeholder partnerships (Sustainable Development Goal 17) and collaborations that mobilize, and share knowledge, expertise, technologies and financial resources could contribute to break down barriers between different stakeholder groups (private, public institutions and civil society), and ultimately better manage complex choices that affect the future of food systems in their necessary transformation.

## Figures and Tables

**Table 1:** Criteria, sub-criteria, and questions included in the interviews.

Criteria	Sub-Criteria	Questions
Economic	Profitability	How profitable do you think this product is? Do you think it is a sector where you can earn a lot of money?
	Stability	How stable do you think this sector is in terms of agricultural prices and yields? Is this sector safe from sudden ups and downs in prices?
	Affordability	Do you think the product is affordable? How much?
Social	Food security	Do you think that this product makes the protein more accessible/available to the consumer?
	Labour conditions	Do you think that the working conditions in this sector are adequate? (salary, labour accidents, and labour laws)
	Supportive of health and nutrition	Do you think that this product is healthy and nutritious?
	Adequate information about the product	Do you think the information available is adequate/sufficient?
Environmental	Emissions related to climate change	Do you think these products have a low greenhouse gas emission rate?
	Use of natural resources (water and land)	Do you think that these products have a low rate of natural resources consumption (land and water)?
Policy	CAP subsidies convenience	Are Common Agricultural Policy (CAP) subsidies convenient?
	Adequate regulation/food legislation (food safety)	Is there adequate regulation/legislation regarding food safety in this sector?
Product Features	Organoleptic properties	Do you think that this product is tasty and pleasant?
	Ease of preparation	Do you think this product is easy to cook?
Technological	Adequate production and processing technology	Do you think that this sector has adequate production and processing technology?

## Appendix A

**Table A1:** Mean criteria and sub-criteria weights for each stakeholder group. Criteria weights are express in absolute values and in percentage terms. Sub-criteria values are expressed in percentage of absolute criteria value.

Criteria (and Sub-Criteria)	Producer	Processor	Distributor	Consumer	Policy Maker	Researcher	NGO	Average	
Economic	82.4	70.1	78.7	83.3	70.4	76.8	79.2	77.3	19.6%
Profitability	34%	31%	27%	29%	24%	30%	28%	29%	
Stability	24%	29%	28%	31%	21%	25%	28%	27%	
Affordability	42%	40%	45%	40%	55%	44%	43%	44%	
Social	63.8	79.3	66.8	75.9	65.6	58.9	70.5	68.7	17.4%
Food Security	19%	23%	22%	26%	18%	18%	25%	22%	
Labor conditions	29%	21%	16%	23%	18%	21%	25%	22%	
Health and nutrition	33%	35%	36%	28%	42%	37%	26%	34%	
Product information	19%	21%	26%	23%	21%	24%	24%	22%	
Environmental	66.5	73.7	58.7	80.2	74.4	63.6	84.2	71.6	18.2%
GHG emissions	44%	49%	41%	50%	44%	51%	47%	47%	
Natural resources	56%	51%	59%	50%	56%	49%	53%	53%	
Policy	44.8	40.4	54.9	39.4	64.4	56.9	61.4	51.7	13.1%
CAP support	47%	37%	28%	42%	33%	47%	53%	41%	
Food legislation	53%	63%	72%	58%	67%	53%	47%	59%	
Product features	69.8	84.1	78.9	68.4	74.3	72.3	78.2	75.1	19.0%
Organoleptic properties	67%	54%	72%	58%	71%	62%	69%	65%	
Cooking properties	33%	46%	28%	42%	29%	38%	31%	35%	
Technological	55.4	45.8	62.3	25.7	54.5	52.7	53.2	49.9	12.7%

**Table A2:** Performance of food products under each criterion by stakeholder group (average weight of each criterion (%).

Stakeholders	Food Product	Economic	Social	Environmental	Policy	Product Features	Technological
**Producer**	Chicken meat	21%	16%	15%	12%	20%	16%
	Dairy milk	18%	17%	12%	13%	22%	17%
	Plant-based meat	19%	14%	20%	13%	19%	15%
	Plant-based milk	17%	15%	19%	13%	20%	15%
**Processor**	Chicken meat	20%	19%	11%	10%	26%	14%
	Dairy milk	18%	21%	12%	11%	26%	13%
	Plant-based meat	18%	18%	18%	11%	22%	13%
	Plant-based milk	18%	17%	17%	11%	24%	13%
**Distributor**	Chicken meat	18%	16%	12%	16%	24%	14%
	Dairy milk	17%	16%	11%	15%	25%	16%
	Plant-based meat	17%	16%	15%	14%	25%	14%
	Plant-based milk	18%	16%	15%	15%	22%	15%
**Consumer**	Chicken meat	25%	20%	11%	11%	24%	9%
	Dairy milk	25%	21%	11%	10%	24%	10%
	Plant-based meat	19%	19%	24%	12%	18%	7%
	Plant-based milk	19%	20%	23%	12%	20%	6%
**Policy Maker**	Chicken meat	20%	16%	11%	17%	22%	15%
	Dairy milk	17%	16%	9%	20%	23%	15%
	Plant-based meat	14%	15%	22%	18%	17%	14%
	Plant-based milk	15%	15%	21%	19%	16%	14%
**Researcher**	Chicken meat	21%	16%	11%	14%	21%	17%
	Dairy milk	19%	15%	9%	16%	22%	18%
	Plant-based meat	17%	14%	16%	18%	20%	15%
	Plant-based milk	17%	14%	15%	19%	20%	16%
**NGO**	Chicken meat	22%	17%	12%	14%	22%	14%
	Dairy milk	20%	17%	10%	15%	25%	14%
	Plant-based meat	16%	15%	20%	16%	20%	12%
	Plant-based milk	18%	16%	22%	17%	15%	13%
**All**	Chicken meat	21%	17%	12%	13%	23%	14%
	Dairy milk	19%	17%	10%	14%	24%	15%
	Plant-based meat	17%	16%	19%	15%	20%	13%
	Plant-based milk	17%	16%	19%	15%	19%	13%

**Table A3:** Performance of animal-based and plant-based products under each sub-criterion by stakeholder group (average weight of each sub-criterion in percentage).

Stakeholder	Food Product	Economic			Social				Environmental		Policy		Product Features		Technological
		Profitability	Stability	Affordability	Food Security	Labor Conditions	Health, Nutrition	Product Information	GHG Emissions	Natural Resources	CAP Support	Food Legislation	Organol. Propert.	Cooking Propert.	Technology
**Policy Maker**	Animal-based	46%	50%	56%	54%	49%	48%	52%	38%	39%	44%	50%	54%	50%	50%
	Plant-based	54%	50%	44%	46%	51%	52%	48%	62%	61%	56%	50%	46%	50%	50%
**Consumer**	Animal-based	47%	46%	63%	47%	46%	46%	51%	29%	29%	37%	46%	52%	52%	55%
	Plant-based	53%	54%	37%	53%	54%	54%	49%	71%	71%	63%	54%	48%	48%	45%
**Distributor**	Animal-based	46%	56%	54%	58%	46%	48%	53%	45%	45%	61%	51%	57%	50%	53%
	Plant-based	54%	44%	46%	42%	54%	52%	47%	55%	55%	39%	49%	43%	50%	47%
**NGO**	Animal-based	47%	49%	59%	54%	44%	45%	56%	33%	31%	40%	48%	57%	50%	51%
	Plant-based	53%	51%	41%	46%	56%	55%	44%	67%	69%	60%	52%	43%	50%	49%
**Processor**	Animal-based	44%	50%	58%	56%	46%	52%	61%	38%	39%	47%	50%	56%	51%	50%
	Plant-based	56%	50%	42%	44%	54%	48%	39%	62%	61%	53%	50%	44%	49%	50%
**Producer**	Animal-based	42%	50%	63%	62%	48%	54%	56%	41%	43%	51%	50%	57%	50%	54%
	Plant-based	58%	50%	37%	38%	52%	46%	44%	59%	57%	49%	50%	43%	50%	46%
**Researcher**	Animal-based	50%	50%	59%	58%	45%	48%	59%	36%	39%	38%	50%	53%	50%	52%
	Plant-based	50%	50%	41%	42%	55%	52%	41%	64%	61%	62%	50%	47%	50%	48%
**All**	Animal-based	46%	50%	58%	55%	46%	49%	55%	37%	38%	45%	49%	55%	50%	52%
	Plant-based	54%	50%	42%	45%	54%	51%	45%	63%	62%	55%	51%	45%	50%	48%
